# Group Living Enhances Individual Resources Discrimination: The Use of Public Information by Cockroaches to Assess Shelter Quality

**DOI:** 10.1371/journal.pone.0019748

**Published:** 2011-06-20

**Authors:** Stéphane Canonge, Jean-Louis Deneubourg, Grégory Sempo

**Affiliations:** Unit of Social Ecology, Université libre de Bruxelles (ULB), Brussels, Belgium; Cajal Institute, Consejo Superior de Investigaciones Científicas, Spain

## Abstract

In group-living organisms, consensual decision of site selection results from the interplay between individual responses to site characteristics and to group-members. Individuals independently gather personal information by exploring their environment. Through social interaction, the presence of others provides public information that could be used by individuals and modulates the individual probability of joining/leaving a site. The way that individual's information processing and the network of interactions influence the dynamics of public information (depending on population size) that in turn affect discrimination in site quality is a central question. Using binary choice between sheltering sites of different quality, we demonstrate that cockroaches in group dramatically outperform the problem-solving ability of single individual. Such use of public information allows animals to discriminate between alternatives whereas isolated individuals are ineffective (i.e. the personal discrimination efficiency is weak). Our theoretical results, obtained from a mathematical model based on behavioral rules derived from experiments, highlight that the collective discrimination emerges from competing amplification processes relying on the modulation of the individual sheltering time without shelters comparison and communication modulation. Finally, we well demonstrated here the adaptive value of such decision algorithm. Without any behavioral change, the system is able to shift to a more effective strategy when alternatives are present: the modification of the spatio-temporal distributions of individuals leading to the collective selection of the best resource. This collective discrimination implying such parsimonious and widespread mechanism must be shared by many group living-species.

## Introduction

One of the aims of collective behavior study [1] is to understand the role of various factors including the presence of group-members as a major influence in regulating the individual decision-making process. Earlier work has shown that consensual decision results from the interplay between individual responses to site characteristics and to group-members [1]–[3]. To go further, it becomes essential to determine the impact of conspecifics in the accuracy of individual's action. In other words, how each individual could increases its own chances of making a correct decision between several alternatives. Indeed, when choosing habitat in patchy environment, group-living species are confronted with a choice between many sites offering the same habitat but differing in their intrinsic quality. There are different ways to make choice: it could refer to high-level of cognitive skills (e.g. distant perception of the two patches, memory and direct comparison between sites). Moreover, social information can provide a more accurate estimate of habitat quality (i.e. improve the correctness of its personal information) [4], [5]. The presence of conspecifics provides a local social cue [6], [7] that can be used by individuals in their ‘shared information’ strategy (i.e. social attraction) [8]–[10]. This source of information is known as public information [11], [12] and is acquired by witnessing the behavioral decisions of other individuals. Here, we study the case where the decision is to stay or not in the patch, individual only uses personal and public information, which are local in time (no memory) and space (no distant perception) (Canonge 2009). If social information only informs about the location of a resource, public information also brings knowledge about its quality [12]. Moreover, the way animals use public information may be influenced by population density [13], [14]. Few studies, however, focus on the gain of individual choice accuracy with group size or population density [5], . In this study the main issue is how population size (i.e. the number of conspecifics or density) modulates the discrimination efficiency between two patches of different quality. This investigation falls within the scope of habitat selection theory. We highlight the mechanisms by which swarm intelligence [17] based on conspecifics' inter-attraction can increase individual fitness (for review [1]). Here, we report an experimental and theoretical study of cockroach behaviors (*Periplaneta americana*) tested in a one-meter-diameter arena with two shelters differing in darkness. As cockroaches have an adaptive interest in avoiding light [18], [19], a dark shelter (75 lx) constitutes a better resting site than a lighter one (100 lx) [20]. To enlighten the specific impact of population size, we had to examine both individual (isolated individual) and collective responses (groups of 10, 16 and 30 cockroaches). We defined the discrimination efficiency as the ratio between the number of individuals under the dark shelter and the number under the light one. In case of isolated individual (i.e. the personal discrimination efficiency), the number of individuals corresponds to the number of experiments for which the individual is respectively under the dark or the light shelter. In case of groups (i.e. the collective discrimination efficiency), it is the number of individuals under dark and under the light shelter. In our experimental set-up, without any a priori information about resources (i.e. shelters), naive individuals have the choice between staying in a shelter and leaving it in quest of a potential better one. This ‘cockroach-shelter’ system is well adapted for the study of mutual benefits between individual and collective decision-making because it provides interplay between mutually exclusive choices and cooperation through individuals' aggregation.

## Results

**Figure 1:**
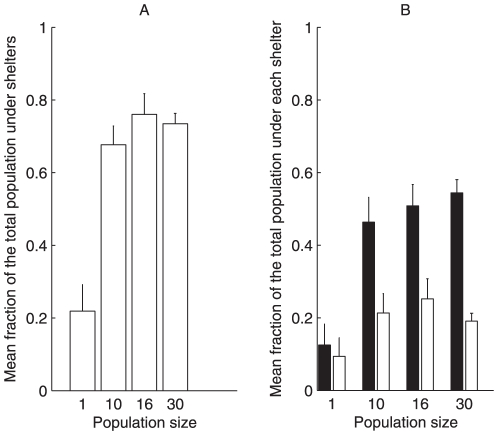
Settlement under shelters according to population size. (1 (n = 32), 10 (n = 30), 16 (n = 30) and 30 cockroaches (n = 25)) at t = 180 min. (A) Mean fraction of the total population under both shelters (Kruskal-Wallis test: KW = 23.6, *p*<0.0001; Dunn's Multiple Comparisons Test: paired comparison including 1 individual *p*<0.01; for other comparisons *p*>0.05); (B) Mean fraction of the total population under each shelter: dark (black) and light (white) (Kruskal-Wallis test for dark shelter: KW = 31.17, *p*<0.0001; Dunn's Multiple Comparisons Test: paired comparison including 1 individual *p*<0.001, for other comparisons *p*>0.05). Error bars indicate S.E.M.

The first evidence about the population size influences was underlined by the cockroach propensity to shelter. Indeed, less than 22% of isolated cockroaches are found under shelters after 180 min (*n* = 32) whereas more than 70% of the total population is sheltered when cockroaches are in groups of 10 (*n* = 30), 16 (*n* = 30) or 30 (*n* = 25) individuals (fig. 1A).

Secondly, we highlighted that the presence of conspecifics enhances the personal discrimination efficiency between two shelters of different darkness. Indeed, the individual trends of settling under the better shelter, the darkest in our experimental design, rises with the population size, as attested by the increase in fraction of the population aggregated under this shelter (fig. 1B). Only 12% of isolated cockroaches settle under the dark shelter, versus 54%±18% of individuals for groups of 30 cockroaches. An individual has a weak preference for the darkest shelter although statistically, there is no difference between the mean fractions under each shelter (Mann Whitney test, *p* = 0.83, n = 32; [5]) (see Table S1). Thanks to the interactions between group-members, however, a population of cockroaches is more likely to respond to environmental heterogeneities and to aggregate in the better resting site. Indeed, individuals in groups strongly prefer to settle under the dark shelter (for all groups comparison: Mann Whitney test, *p*<0.05) (see Table S1).

**Figure 2:**
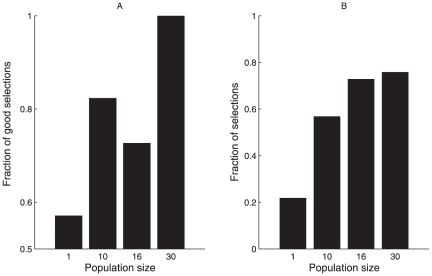
Shelter selection frequency. (A) Fraction of replicates ending with good selection (i.e. selection of the darkest shelter) according to population size ( = discrimination efficiency); (B) Fraction of replicates ending with the selection of one of the two shelters according to population size ( = ability to make a choice) (see SI 2 for statistical criteria of selection).

This is confirmed by the increase of the darkest shelter selection frequency related to the population size (fig. 2A, see also Text S1). Few isolated cockroaches settled under the shelters with a weak preference for the darkest one. On the contrary, for populations of 10 and 16 cockroaches, 53% of replicates ended with the selection of the dark against only 27% and 20% respectively for the light shelter. For a population of 30 cockroaches, the selection of the dark shelter is more marked and reached 76% of replicates while the light shelter was never selected. These results demonstrated that being in a group enhances the capability to select the better shelter.

Based on previous studies showing the role of the interactions between conspecifics on cluster formation and resting site selection [3], [21], [22], we analyzed how the personal discrimination efficiency between sites can be enhanced by the population size. For this, we used a dynamical model of shelter selection process in cockroaches, which has been validated in other contexts [3], [20], [21]. This model describes the dynamical process of collective decision in terms of individual joining or leaving a shelter, depending on its quality and the number of conspecifics that are already there (a full description is given in Text S2). In this model, a cockroach is located either under the dark, the light shelter or outside. Joining is accounted by *R_D_* and *R_L_* as the probabilities per second (s^−1^) of joining the dark or the light shelter respectively, and leaving is accordingly accounted by ,*Q_D_* and *Q_L_*. The probabilities of joining are given by:
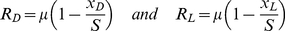
(1)where 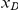 (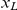) is the number of cockroaches under the dark (light) shelter. Parameter *μ* represents the maximal kinetic constant of joining a shelter. As the probability of joining a shelter is independent of its luminosity (this is due to the lack of distant perception), *μ* is equal for each shelter [23]. The shelter carrying capacity *S* corresponds to the number of individuals that can rest simultaneously under the same shelter (see Text S2). Previous studies show that the probabilities of leaving decrease (or the sheltering time increases) with the number of individuals in the same shelter [3], [21]. These probabilities are given by:
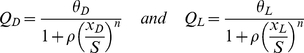
(2)Parameters *ρ* and *n* refer to the strength of the affinity between conspecifics and correspond to the implementation of the social information. When we have no social interaction, *ρ* = 0 and *n* = 0. In this situation, the probability of leaving a shelter is independent of the sheltered population. The parameter *θ* depends on shelter quality (i.e. light intensity) with *θ_L_*>*θ_D_*. The ratio between the personal probabilities of leaving the light∶dark shelter is defined as the personal discrimination power: 
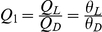
 (increasing from 1 when no discrimination to values >1 when discrimination between shelters). The parameter values (*μ*, *ρ*, *θ*, *n* and S) are derived from both experimental data and previous estimation [20], [22], [23] (see Text S2).

**Figure 3:**
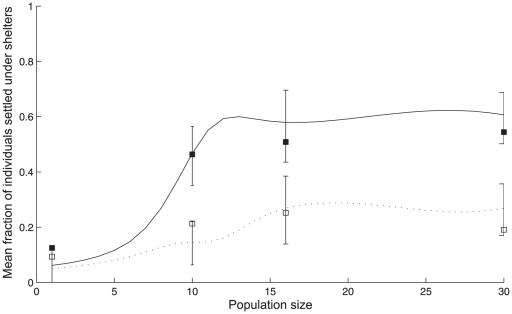
Theoretical and experimental discrimination efficiency. The resolution of the numerical equation gives the mean fraction of individual settled under the dark (solid line) and the light (dashed line) shelter for population size varying between 1 and 30 individuals (*θ_D_* = 0.22, *θ_L_* = 0.27). Only for values corresponding to experimental population (1, 10, 16 and 30 individuals), we draw the confidence intervals (vertical lines) containing 95% of the mean theoretical results for groups of *n* experimental replicates (see statistical analysis section). The experimental results (full squares for dark and open squares for light) fall within the confidence intervals of the theoretical data.

We assumed that these observed aggregation (fig. 2A and 2B) patterns result from a weak individual preference for the darkest shelter strongly amplified through the modulation of individual sheltering times. To test this hypothesis, we performed numerical resolution of differential equation (see master equation *eq.3*) under the assumption that the state of the system (i.e. the number of individuals outside (*i*), under the dark (*j*) and under the light shelter (*k*)), is described in terms of a probability function *P*(*i,j,k*) at the time t (*i+j+k = N*, the total number of individuals). The equation (*eq.3*) describes the time evolution of the probability that the system (*P(i,j,k)*) occupies each one of the discrete set of states. The equation counts the transitions leading the system to certain state and those removing it from this state. In our case, the transitions depend on both the probability of joining and leaving the shelters (see Flowchart S1) and the evolution of the master equation (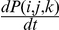) is :
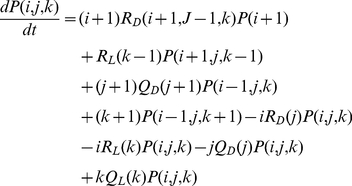
(3)At time t = 0, P(N,0,0) = 1. The accordance between theoretical and experimental results validates our hypothesis. Indeed, both theoretical and experimental discrimination efficiency are similar (fig. 3). For population of at least 10 individuals, the mean fraction of individuals settled under the dark shelter reaches a plateau value (around 60% under the dark shelter and 25% under the light one). Moreover, the theoretical distributions of replicates according to the fraction settled under the dark shelter are in good accordance with the experimental one (see fig. S1), Kolmogorov-Smirnov test for all population size: *p*>0.05). Without global knowledge, cockroaches use public information to reach a consensual decision keeping group cohesion. Moreover, each individual increases its own chances to make a correct decision with population size.

**Figure 4:**
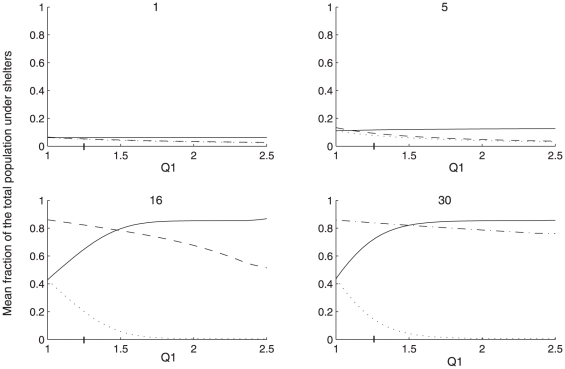
Theoretical results. Comparison between theoretical fractions of individuals settled in a binary choice (a dark shelter (solid line) and a light one (dotted line)) and in a no choice setup (corresponding to the light shelter (dashed line)) according to Q_1_. Vertical bars on x-axis represent the experimental Q_1_.

To go further, we theoretically tested for different population size the influence of a modulated difference between the dark and the light shelter quality. To do so, *θ_D_* was kept constant while the personal discrimination power Q_1_ varies from 1 to 2.*5*. These results reveal that the bigger the population is, the smaller the ratio between shelters quality is needed to lead the group to the selection of the best (dark) shelter. Indeed, for large groups (>16 individuals), 100% of the sheltered population is under the dark shelter. In a no choice setup (one shelter) the fraction found under the light shelter decreases when *θ_L_* increases but remains closed to the fraction under the dark shelter in a binary-choice setup (a dark vs a light shelter, fig. 4).

For isolated or small population (≤5 individuals), the fraction of settled individual under the light and the dark shelter and the collective discrimination efficiency remain small (with or without choice). In other words, bigger population is more accurate to select the best shelter even in case of very small difference between potential resting sites. These theoretical results confirmed that the state of the system is population size dependent.

Without social interaction (*ρ* = 0 or *n* = 0), there is not such a dependence on the population size: the collective discrimination efficiency is always equal to the personal one (see fig. 4 for isolated). Moreover, the model shows that the collective discrimination efficiency is equal to the personal discrimination power (Q_1_), therefore, a high level of discrimination is only reached if Q_1_ is high.

## Discussion

The ability to aggregate in the darkest and most populous shelter is adaptively crucial for cockroaches. Indeed, the benefit of staying in a shelter increases with its darkness (e.g. light has a negative effect on their growth [18], [19]) and owing to several Allee effects it increases with the number of surrounding congeners [24]–[26]. Here we show that the discrimination efficiency between sites and the emerging consensus for the selection of the better one increase with the population size (and reach a plateau value for around 15 individuals). This phenomenon is a by-product of an aggregation dynamics governed by the competition between amplification processes. As cockroaches use limited local information, without any direct comparison between shelter qualities [23], the individual decision to stay under a shelter only relies on its darkness and its number of settled conspecifics. Consequently, the individual probability of correctness increases with the population size. This increase is especially strong and the need of public information is relevant when the quality of the sites does not differ much (here, the difference between both quality is small). The efficiency of this mechanism contrasts with its parsimony. The interactions between individuals are not modulated by the shelter quality, which only affect the individual response (resting time under shelter) [16], [27]. Moreover, the mechanisms allow for better choice at the individual scale when the population is confronted to a choice, but when the choice is not optimal [28], [29] (replicate ending with the selection of the light shelter) it does not prevent to get the benefits of being gathered due to Allee effect (see fig. 2B) [30], [31]). Our results show that if public information includes patch quality, it also indirectly integrates the influence of other patches and population size. Indeed, low populations did not favor settlement and shelter selection and consequently maintained an exploration activity leading to the discovery of a new area potentially containing high population density [32]–[34]. This kind of behavior could be a good strategy at the population level: group-members continue to explore the environment and may discover a better sheltering site. We well demonstrated the adaptive value of such decision algorithm. When only one resource is present, population settled under it. But when a better alternative is present, trough local social interactions between conspecifics, the system shifts to a more adaptive strategy: the selection of the best resource. As in many collective phenomena (e.g. [16], [22], [35], [36]), this mechanism is strongly population size dependent. This result seems in agreement with the theoretical predictions of Rands [37] on the decrease of the effort made by an individual with increasing group size.

Despite the lack of long-range communication (through e.g. vision orientation, trail following), of comparison and of any knowledge of the spatial dispersion of the resources, cockroaches may collectively discriminate between spatially scattered alternatives and select the best one. Previous studies on swarm intelligence and more broadly on population dynamics have reported for several activities or species characterized by different degrees of sociality or cognition that the competition between amplifying communication processes enables them to solve problems that are beyond the individual's capacity [1], [16], [38], [39]. In an evolutionary point of view, we hypothesized that higher-level cognitive species are likely to use the same kind of process to select for the optimal site [16], [35]. Indeed, there is no need that the evolution to new cognitive skills should have erased and replaced the processes that had actually worked so far. Our theoretical model shows that without social amplification (*ρ* = 0 or *n* = 0), i.e. when individuals act independently from each other, settlement and the selection of the best shelter can only occur with strong personal discrimination power.

From a general perspective about the fitness of collective decisions, it is nevertheless the demonstration that such collective discrimination is a by-product of gregarious behavior, the most basic and widely spread social behavior [9]. This suggests that similar collective discrimination processes should be at work in various taxonomic groups and for a large variety of environmental cues (humidity, temperature, chemical landscape,…). Potentialities of gregarious behavior, contrasting with their parsimony, are also illustrated by the optimal responses to the resource limitations. These systems are governed by nonlinear dynamics that, through the individual response to different environmental parameters, favor the difference between individual and collective behavior (e.g. discrimination power vs collective discrimination) and can also lead to a cascade of complex structures [2], [35], [36]. A better understanding of mechanisms is required to understand how the global complexity and functionality of the collective patterns emerge. This knowledge should also enlighten how the natural selection could shape individual performances in gregarious species where the individual capacity to make the good choice takes a part in the collective decision which in turn enhances it. This is especially required for understanding the impact of group size upon individual fitness [3].

## Materials and Methods

### Biological model

Experiments were carried out on adult males (average length: 4 cm) of the cockroach species *Periplaneta americana* (L.) (Dictyoptera: Blattidae). This specie has a worldwide distribution and is closely associated with human dwellings, food-processing industries, and shows dense populations in urban areas. *P. americana* alternates diurnal phases of aggregation inside shelters and nocturnal phases of exploration and foraging [40], [41]. During the day *P. americana* (like most of the gregarious cockroach species) rests in aggregates that include males and females of all stages/ages [40], [42]. Adult males of *P. americana* were reared in transparent boxes (80×40×100 cm) containing shelters (cardboard cylinders: length 30 cm, diameter 5 cm). They had *ad libitum* access to water and food pellets (Tom & Co™ dog food). Cockroaches were kept at a temperature of 25°C±1°C and in a 12 h/12 h light/dark cycle.

### Experimental Procedure [for further see 20], [ 23]

Two days before being tested, adult males of *P. americana* were taken out of the rearing box and isolated for 48 hours in total darkness in a smaller box (36×24×14 cm) containing water, food pellets (Tom & Co™ dog food) and shelters (cardboard cylinders: length 30 cm, diameter 5 cm). After this isolation period, individuals were introduced into the center of a circular arena (375 lx, diameter 1 m) including only a dark (75 lx, diameter 15 cm) and a light (100 lx, diameter 15 cm) shelter. At 180 minutes, the number of individuals under each shelter reached a plateau value and was counted by means of a video camera placed between lamps and centered on the arena.

### Statistic Analyses

Data from all the experiments were tested for any deviance from normality with the Kolmogorov-Smirnov test. When normality conditions were met we carried out parametric tests; otherwise, we performed corresponding nonparametric tests. We applied the Mann-Whitney test to compare the mean fraction of individuals settled under the dark or the light shelter (see Table S1). The deviation from a binomial distribution was used in order to highlight an amplification process in the spatial distribution of individuals (see fig. 2AB and Text S1). In fig. 3, for each population size (1, 10, 16 and 30 individuals), the mean numbers of individuals under the dark and the light shelter for *n* experimental replicates were compared with the means of *n* theoretical replicates. Based on the theoretical probability (*P*(*i,j,k*)) that the system is in a state with *j* individuals under the dark shelter (with *k* individuals under the light shelter), we computed the mean value of *j* (*k*) for groups of n replications. The distribution of this theoretical mean value is well approximated by a Gaussian function. From this distribution we defined a confidence interval containing the 95% most probable averages, with which we compared the experimental one. For Figure S1, we used the Kolmogorov-Smirnov test to determine if the experimental distributions of the fraction of individuals under the dark shelter for each population size differed significantly from the theoretical ones. This test makes no assumption about the distribution of the data. All tests were two-tailed and the significance of all the statistical tests was fixed at α = 0.05.

## Supporting Information

Figure S1 Theoretical and experimental replicate distributions.
(EPS)

Table S1 Experimental fraction under shelters.
(DOC)

Text S1 Statistical criteria for shelter selection.
(DOC)

Text S2 Mathematical model of individual decision.
(DOC)

Flowchart S1 Flowchart illustrating the transition probabilities between the different states of the system.
(EPS)
