# Supplementary material for: Group Living Enhances Individual Resources Discrimination: The Use of Public Information by Cockroaches to Assess Shelter Quality
Source: PLoS One. 2011 Jun 20;6(6):e19748. doi: 10.1371/journal.pone.0019748 (PMC3119082; doi:10.1371/journal.pone.0019748)
Supplement: Table S1 — Experimental fraction under shelters. (DOC) [file pone.0019748.s002.doc]

**Table S1**

| Condition | Fraction of cockroaches under shelters at t = 180 min  (Mean fraction of individuals ± s.d.) | | |
| --- | --- | --- | --- |
|  | Dark | Light | *Mann-Whitney test* |
| 1 cockroach  (n = 32) | 0.12 ± 0.34 | 0.09 ± 0.30 | *P = 0.83* |
| 10 cockroaches  (n = 30) | 0.46 ± 0.38 | 0.21 ± 0.30 | *P < 0.05* |
| 16 cockroaches  (n = 30) | 0.51 ± 0.33 | 0.25 ± 0.31 | *P < 0.001* |
| 30 cockroaches  (n = 25) | 0.54 ± 0.18 | 0.19 ± 0.11 | *P < 0.0001* |

**Table S1.** **Experimental fraction under shelters:** Comparison between the mean fraction under the dark and the light shelter at *t* = 10 800s for each population size.
