# Supplementary material for: Group Living Enhances Individual Resources Discrimination: The Use of Public Information by Cockroaches to Assess Shelter Quality
Source: PLoS One. 2011 Jun 20;6(6):e19748. doi: 10.1371/journal.pone.0019748 (PMC3119082; doi:10.1371/journal.pone.0019748)
Supplement: Text S1 — Statistical criteria for shelter selection. (DOC) [file pone.0019748.s003.doc]

**Text S1**

**Statistical criteria for shelter selection**

Assuming first that cockroaches settlement in a shelter is neither influenced by its luminosity [23] nor by the number of congeners it contains and second that the probabilities of entering in the dark (*d*) or light (*l*) shelter are equal (*p_d_* = *p_l_* = 0.5), we can calculate the probability (*P*) of observing a replicate ending with *i* individual in one of the two shelters. The expected distribution of individual under each shelter should then fit the following binomial function:

$$P(N,i)=\frac{N!}{i!(N-i)!}0.5^{N}$$

where *N* corresponds to the total number of individuals in the two shelters and *i* is the number of individuals under one of the two shelters. The *p* value gives us the probability that the observed spatial distribution of individuals between the two different shelters is owed to a random distribution. A shelter was considered as being selected by the group when *p* > 0.05, which corresponds to a statistical difference between the observed and the expected distribution of sheltered individuals.
