# Supplementary material for: Group Living Enhances Individual Resources Discrimination: The Use of Public Information by Cockroaches to Assess Shelter Quality
Source: PLoS One. 2011 Jun 20;6(6):e19748. doi: 10.1371/journal.pone.0019748 (PMC3119082; doi:10.1371/journal.pone.0019748)
Supplement: Text S2 — Mathematical model of individual decision. (DOC) [file pone.0019748.s004.doc]

**Text S2**

**Mathematical model of individual decision**

**- Decision probabilities**

This model is based on observations showing that no long-range interaction among cockroaches occurs [3, 20, 21]. Neither chemical marking nor memory effect had a significant effect because, in our experimental conditions, the cockroaches were placed for a short time (180 min) in a new environment free of chemical traces laid by conspecifics and were surrounded by a uniform enclosure, preventing cockroaches from using spatial elements beyond the set-up. We considered the following experimental facts: (i) individuals explore their environment randomly and thus encounter shelters randomly independently of their light intensities [23]; (ii) they rest in shelters according to their light intensities [3, 23]; and (iii) they are influenced by the presence of conspecifics through social amplification of resting times [3, 20, 21], all individuals being considered equal. This model has been partly validated for two species of cockroaches (*Blattella germanica* [3], *Periplaneta Americana* [20]), for juveniles and adults and for males and females.

The probability of joining (*R_D_* and *R_L_*) and the probability leaving (*Q_D_* and *Q_L_*) are given by:

$$R_{D}=\mu\left(1-\frac{x_{D}}{S}\right) and R_{L}=\mu\left(1-\frac{x_{L}}{S}\right) (eq.1)$$

$$Q_{D}=\frac{\theta_{D}}{1+\rho\left(\frac{x_{D}}{S}\right)^{n}} and Q_{L}=\frac{\theta_{L}}{1+\rho\left(\frac{x_{L}}{S}\right)^{n}} (eq.2)$$

where *x_D_* (*x_L_*) is the number of individuals under dark (light) shelter. The parameter *θ* is the maximal rate of leaving a shelter according to its light intensity. The ratio between the personal probabilities of leaving the light : dark shelter is defined as the personal discrimination power: $Q_{1}=\frac{Q_{L}}{Q_{D}}=\frac{\theta_{L}}{\theta_{D}}$ (increasing from 1 when no discrimination to values > 1 when discrimination between shelters). Based on the comparison between our simulations and our experiments, the best fits obtained for the dark shelter was *θD* = 0.22 s^-1^ and for the light *θL* = 0.27 s^-1^. Our ratio between *θL/ θ*D is = 1.23 and is close to those estimated [20,23] (around 1.2 – 1.4). Moreover, our value of *θL* (*θD*) is close to the mean between the values of *θL* (*θD*) given by Halloy *et al.* (2007) and by Canonge *et al.* (2009). These mean values are *θL* = 0.28 s^-1^ and for *θD* = 0.20 s^-1^. The parameters *ρ* and *n* take into account the influence of the cockroaches’ conspecifics, here *ρ* = 4,194 and *n* = 2 [20]. The term *µ* represents the maximal kinetic constant of entering shelter and equals 0.0027s^-1^. The carrying capacity *S* (i.e. the maximum number of cockroaches that can be hosted in the shelter) can be estimated by the ratio between the shelter area (176.71cm^2^) and the average cockroach area (± 6cm^2^) and corresponds to *S* = 30.

**- Master Equation**

The master equation, a set of first-order differential equations, describes the time evolution of the probability of the system to occupy each one of the discrete sets of states (see flowchart S1). *P(U)* is the probability for the system to be in state *U*. The equation counts the processes leading the system to a certain sate *U* and the processes removing it from this state:

$$\frac{dP(U)}{dt}=V^{+}-V^{-}$$

where *V^+^* is the contribution of transitions to state *U* per unit time and *V^-^* is the contribution from state *U* per unit time

It expresses the rate V^+^ as the product of transition probability per unit time of going from state *U’* to *U*, times the probability of being in the state *U’* at time *t*, summed over all states *U’* that can lead to *U* in a single step. Similarly, *V^-^* is the product of the probability of being in state *U* at time *t*, times the sum of the transition probabilities per unit time from *U* to all states *U’* accessible from *U*. In our case, *U* is a state of the system *(i,j,k)* where *i* individuals are locate outside the shelters, *j* under the dark and *k* under the light shelter with (*i,j,k* = 0,…,N). The time evolution $\frac{dP(i,j,k)}{dt}$ is given in terms of birth and death processes. The four transitions leading to the state (*i,j,k*) (i.e. birth processes are (see Flowchart S3):

**Flowchart S3** Flowchart illustrating the transition probabilities between the different states of the system (birth terms: black arrows, death terms: black dotted arrows). *i*, *j* and *k* represent respectively the number of individuals located outside, in the dark or in the light shelter.

*i-1,j+1,k → i,j,k* => one of the *j+1* cockroaches under the dark shelter leaves.

*i-1,j,k+1 → i,j,k*  => one of the *k+1* cockroaches under the light shelter leaves.

*i+1, j-1,k → i,j,k* => one of the *i+1* cockroaches outside the shelters joins the dark shelter.

*i+1,j,k-1 → i,j,k*  => one of *i+1* cockroaches outside the shelters joins the light shelter.

Similarly, there are four death terms:

i,j,k *→ i+1,j-1,k*

i,j,k *→ i+1,j,k-1*

i,j,k *→ i-1,j+1,k*

i,j,k *→ i-1,j,k+1*

To each birth and death term, it corresponds a transition probability, equal to the individual probability times the number of individuals able to perform the corresponding behavior. For example, the transition probability (*P_t_*) between (*i+1,j-1,k*) and (*i,j,k)* is equal to the individual probability per unit of time of joining the dark shelter $R_{D}(j)=\mu(1-\frac{j-1}{S})$times the number of potential joiners (*i+1*).

Summarizing, we can write the following equation:

$$\begin{matrix} \frac{dP(i,j,k)}{dt}=(i+1)R_{D}(i+1,J-1,k)P(i+1)+R_{L}(k-1)P(i+1,j,k-1) \\ +(j+1)Q_{D}(j+1)P(i-1,j,k)+(k+1)P(i-1,j,k+1)-iR_{D}(j)P(i,j,k) (eq.3) \\ -iR_{L}(k)P(i,j,k)-jQ_{D}(j)P(i,j,k)+kQ_{L}(k)P(i,j,k) \end{matrix}$$

The initial conditions at *t* = 0 are *P(N,0,0)* = 1 and *P(i,j,k)* = 0 for *i* different from N, and *j,k* > 0.
